# Atraumatic Restoration of Vertical Food Impaction with an Open Contact Using Flowable Composite Resin Aided by Cerclage Wire under Tension

**DOI:** 10.1155/2016/4127472

**Published:** 2016-08-07

**Authors:** Quan-Li Li, Chris Ying Cao, Qiang-Jian Xu, Xiao-Hua Xu, Jia-Li Yin

**Affiliations:** Department of Prosthodontics, College of Stomatology, Anhui Medical University, Hefei 230032, China

## Abstract

To date, treating vertical food impaction with open contact effectively, especially with an atraumatic therapy, remains a challenge. In this study, we developed a simple, atraumatic, and economic therapeutic measure to treat vertical food impaction. The scientific rationale of our therapeutic technique is to restore an intact and firm proximal contact with proper location and form relationships to prevent forceful interproximal wedging of food, which in turn protects interdental papilla. We performed the procedure using flowable composite resin or composite resin cement with the aid of a cerclage wire under tension to rebuild the contact area. The reported method is especially useful for some challenging clinical cases, such as food impaction after crown and inlay on onlay restoration, and some conventional treatment methods, such as contouring the marginal ridge and developmental grooves, are ineffective.

## 1. Introduction

Food impaction is the occlusal force wedging of food into interproximal periodontal tissue and is classified into vertical and horizontal [1]. Horizontal food impaction is characterized by lateral pressure from lips, cheeks, and tongue, forcing food dregs and fibers into the enlarged interproximal gingival embrasure for gingival recession caused by all kinds of periodontal disease. This situation is relatively more tolerable compared with vertical food impaction because the food dregs can be easily removed using a dental floss or a tooth pick in gingivoocclusal direction, giving a momentary relief. At present, horizontal impaction is usually remedied with removable prosthetics including hard splint and soft artificial gingival [2]. By contrast, vertical food impaction often results in acute papilla gingivitis and gingival abscess, which is often difficult to endure and usually requires a visit to the dentist [3].

The cause of vertical food impaction is relatively complex. The treatment for vertical food impaction often corresponds to its reason, but the results are often nonperspectives. Occlusal step deformity between marginal ridges of the adjacent teeth and plunger cusp caused by occlusal were some of the possible causes of vertical food impaction. According to the abovementioned reason, the treatment usually includes the contour of the marginal ridge and developmental grooves, the contour of the facial and lingual surfaces by occlusal adjustment, or leveling of the occlusal height of the marginal ridge, as well as recarving of the obliterated fossa to a shallow, saucer-shaped fossa and groove to facilitate food escape (creating food escape groove adjacent to marginal ridge) [2, 4]. Loss of proximal contact (open contact) is the most common reason of vertical food impaction. The abovementioned types of vertical food impaction often finally result in open contact. The other possible reasons for loss of proximal contact include occlusal force causing tooth migration, proximal caries, and improper restoration, such as extraction of the maxillary third molar causes gradual shifting of adjacent tooth, thus creating open proximal contact between maxillary first and second molars, adjacent teeth oblique drift due to nonreplacement of a missing tooth, habits forcing teeth out of position, and periodontal disease. Food impaction associated with open contact causes more probing depth and clinical attachment loss interproximally than that associated with uneven marginal ridge. Treatment of vertical food impaction caused by open contact often relays on the use of fixed prosthetics including inlay, onlay, crown, and splint according to the cause of food impaction [2, 4].

However, apart from injury to sound tooth tissue leading to dentin hypersensitivity, all methods above occasionally fall short, causing frustration to dentists and patients alike. To date, treating vertical food impaction effectively, especially with an atraumatic therapy, remains a challenge. Thus, in this study, we developed a simple and atraumatic technique to treat patients who suffer from vertical food impaction.

## 2. Materials and Methods

### 2.1. Patient Inclusion Criteria

*Inclusion Criteria*. Adult patients, suffering from vertical food impaction for the reason of open contact (defined by unrestricted passage of unwaxed dental floss through the interproximal area) were included in the study. All patients had at least one open contact area between second premolar and first molar or between first molar and second molar of posterior teeth. Patients who fulfilled the inclusion criteria and agreed to participate in the 6-month duration of the study signed an informed consent.

The food impaction involved teeth between natural tooth and natural tooth, natural tooth and prosthesis, silica-based ceramic prosthesis and silica-based ceramic prosthesis, or silica-based ceramic prosthesis and other material prostheses.

*Exclusion Criteria*. Cases of loose tooth or the food impaction involved teeth between metal prosthesis and metal prosthesis, metal prosthesis and aluminum/zirconium ceramic prosthesis, and aluminum/zirconium ceramic prosthesis and aluminum/zirconium ceramic prosthesis were excluded.

### 2.2. Step-by-Step Procedure

#### 2.2.1. Prelude

*Eliminating Other Evident Causes of Food Impaction*. Prior to the procedure, the evident causes of vertical food impaction must be eliminated or relieved through grinding, such as enlarging the buccal and lingual embrasure, creating a food escape groove adjacent to the marginal ridge, leveling the occlusal height of the marginal ridge, and plunger cusp.

*Evaluating the Contact Area.* The contact area must be evaluated thoroughly based on its tightness, location, width, and height.

#### 2.2.2. Selecting the Stainless Wire for Tension around the Contact Area

**Figure 1:**
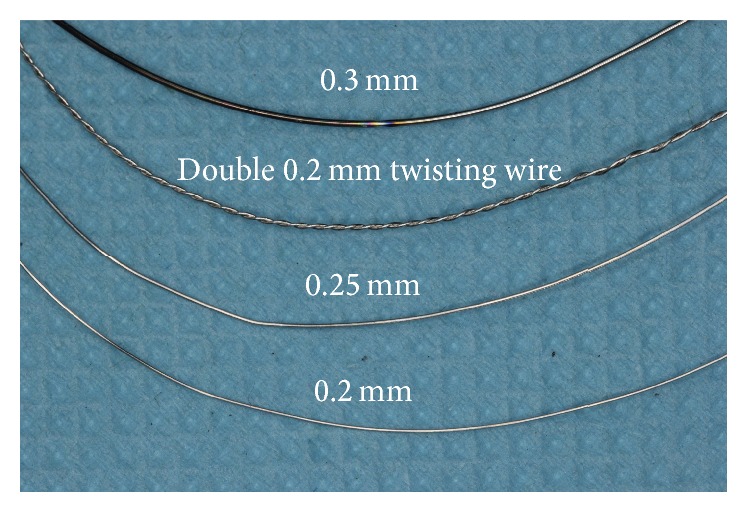
Selecting the stainless wire for tension around the contact area. 0.2, 0.25, or 0.3 mm stainless wire or double 0.2 mm twisting wire can be chosen according to the mesiodistal distance of the contact area. The double-twist 0.2 mm wire should be softened by heat treatment to reduce the rigidity.

We selected 0.2, 0.25, or 0.3 mm stainless wire, or double-twist 0.2 or 0.25 mm wire, which is often used in orthodontic clinics, according to the mesiodistal distance of the contact area (Figure 1). The double-twist 0.2 or 0.25 mm wire and the 0.3 mm wire were softened by heat treatment to reduce their rigidity.

**Figure 2:**
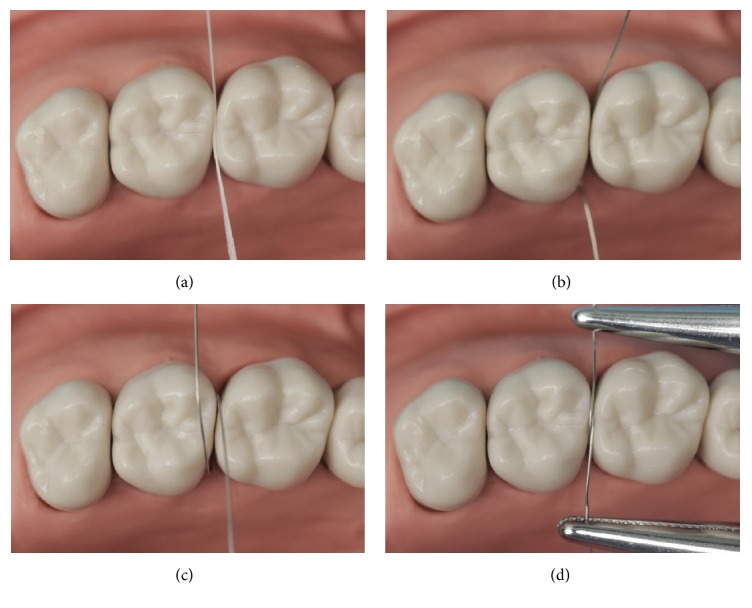
Selecting the stainless wire for tension around the contact area. (a) Evaluating the contact area. Unwaxed dental floss passed through the interproximal area without resistance (open contact). (b) The selected wire passed through the gingival embrasure from buccal surface to lingual surface. (c) Both ends of the wire encircled the contact area and crossed along the occlusal buccal-lingual embrasure. (d) Two hemostatic forceps were clamped to both ends of the wire to enforce buccal-lingual tensile stress, which can separate the teeth appropriately.

The selected wire passed through the gingival embrasure from buccal surface to lingual surface (Figure 2(b)), and then both ends of the wire encircled the contact area and crossed along the occlusal buccal-lingual embrasure (Figure 2(c)). Two hemostatic forceps were clamped to both ends of the wire to enforce buccal-lingual tensile stress (Figure 2(d)). A large force was required for the tension to separate the teeth. The position and form of the area encircled by the wire, which represents the contact to be, were evaluated; if they were inappropriate, then the size of the wire was changed.

#### 2.2.3. Acid-Etching of the Contact Area under Tension Stress with the Cerclage Wire

**Figure 3:**
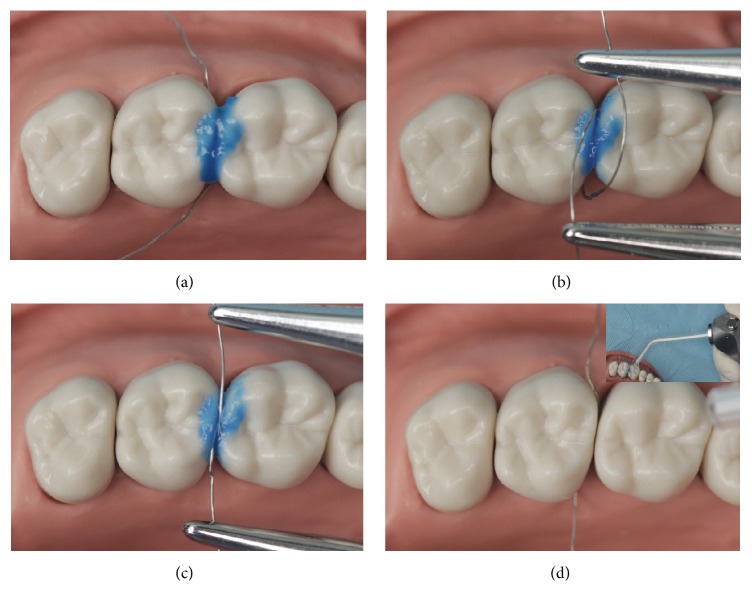
Acid-etching of the contact area under tension stress with the cerclage wire. (a) Applying 20% or 37.5% H_3_PO_4_ gel around the contact area. ((b), (c)) The tension band was enforced with the cerclage wire by squeezing the acid agent into the contact area for 20 s. (d) Rinsing the acid agent thoroughly with water.

H_3_PO_4_ gel (20% or 37.5%) was applied around the contact area of the natural teeth contact or the natural tooth and artificial crown contact (Figure 3(a)). The tension band was then enforced with the cerclage wire by squeezing the acid agent into the contact area for 20 s (Figures 3(b) and 3(c)). Finally, the acid agent was thoroughly rinsed with water (Figure 3(d)).

For contact between silica-based ceramic prostheses, we used 10% HF gel to etch the contact area for 60 s.

#### 2.2.4. Applying Flowable Composite Resin or Composite Resin Cement to Rebuild the Contact Area under Tension Stress with the Cerclage Wire

**Figure 4:**
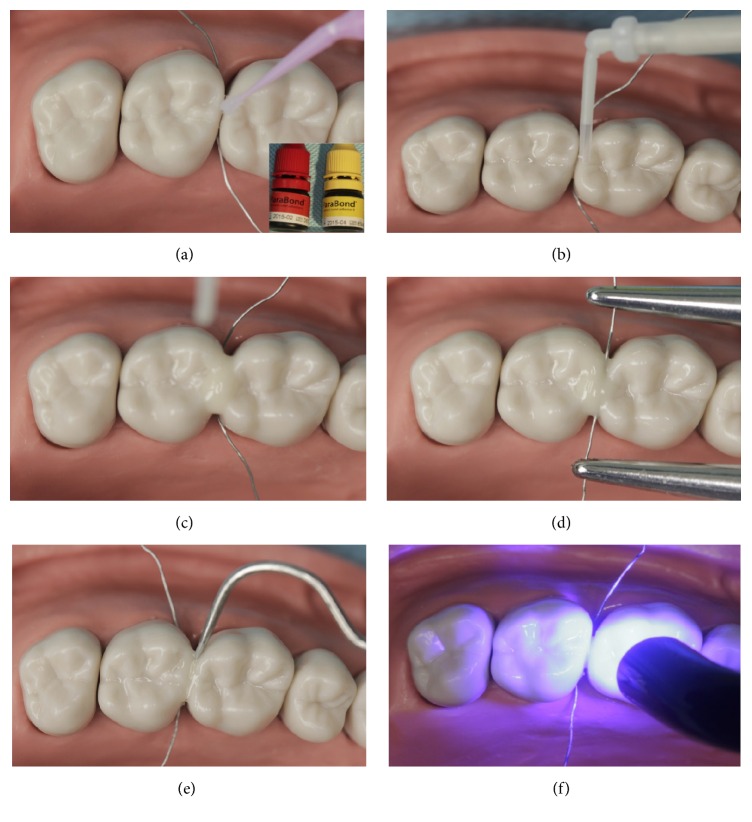
Applying flowable composite resin or composite resin cement to rebuild the contact area under tension stress with the cerclage wire. (a) Applying the primer and bond resin to the etched surface. ((b), (c)) Injecting the flowable composite resin or composite resin cement around the contact area. (d) Enforcing the tension band with the cerclage wire for about 30 s to squeeze the paste of composite resin into the contact area. (e) Relieving the tensile stress and removing the redundancy immediately with a point probe. (f) Light curing from all aspects.

The primer and/or bond resin was applied to the etching surface following the manufacturer's instruction (Figure 4(a)). The flowable composite resin (Tetric® N-Flow, Ivoclar Vivadent AG, Schaan, Liechtenstein) or composite resin cement (ParaCore®, COLTENE, Coltène/Whaledent AG, Altstätten, Switzerland) was then injected around the contact area (Figures 4(b) and 4(c)), enforcing the tension band with the cerclage wire as described above for about 30 s to squeeze the paste of composite resin into the contact area (Figure 4(d)). The tensile stress was then relieved, and redundancy was removed immediately with a point probe (Figure 4(e)). The restoration was polymerised by light curing from all aspects: occlusal, buccal, and lingual surfaces (Figure 4(f)).

#### 2.2.5. Occlusal Adjustment, Polish, and Finish of the Restoration

**Figure 5:**
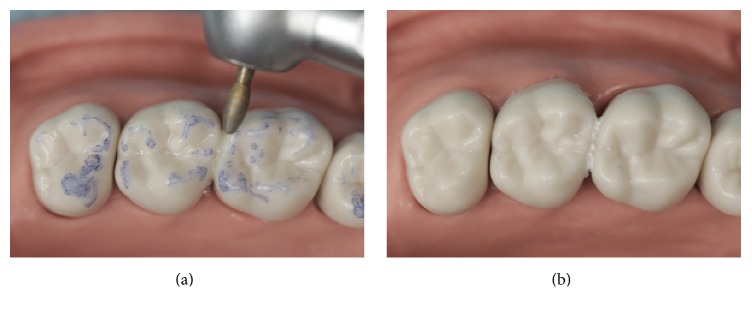
Occlusal adjustment, polish, and finish of the restoration.

Occlusal adjustment was conducted to remove all occlusal interferences (Figure 5). Polish was initiated with prepolish and high shine porcelain silicone points, and definitive polish and high luster were accomplished with a soft hair brush. The final restoration was ensured when the dental floss could not pass through the contact area.

### 2.3. Typical Case

**Figure 6:**
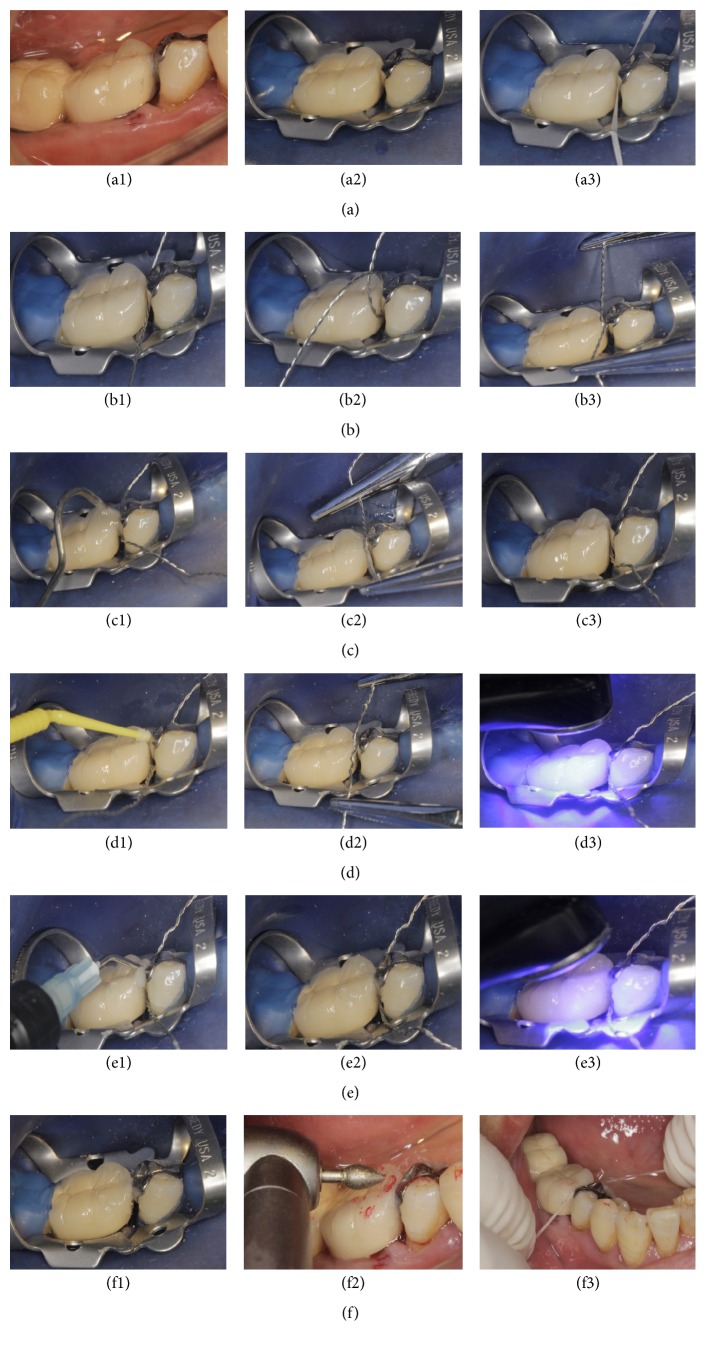
The steps of treatment for food impaction at the right mandible teeth in a typical case. (a) Evaluating the contact area by unwaxed dental floss. (b) Selecting the stainless wire for tension around the contact area. (c) Using 10% HF gel to etch the open contact area. (d) Applying the primer and bond resin to the etched surface. (e) Injecting the flowable composite resin or composite resin cement around the contact area and light curing. (f) Occlusal adjustment, polish, and finish of the restoration.

**Figure 7:**
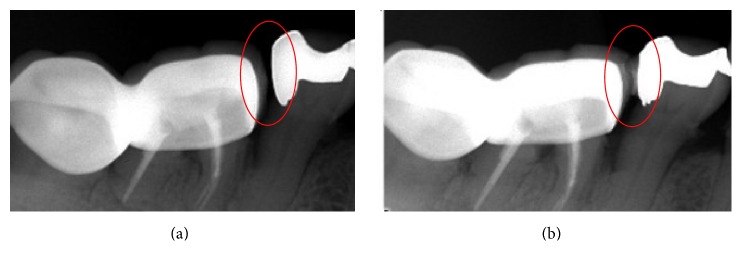
X-ray diagnosis of the interproximal contact area before (a) and after (b) the treatment.

A 65-year-old female complained of food impaction at the right mandible teeth for about 6 months. Oral examination showed that the right mandibular first molar was restored with porcelain-fused-to metal crown, and the right mandibular second premolar was restored with a distal-occlusal metal inlay. The gingival papilla between the second premolar and the first molar in the right mandible receded. Unwaxed dental floss was easily passed through the interproximal area between the two teeth showing an open contact area. The open contact area was etched with 10% HF gel and reconstructed following the method as shown in Figures 6 and 7.

## 3. Results

Thirteen patients, aged 46 to 76 years, suffering from vertical food impaction, were selected. Of them, sixteen open contact areas between second premolar and first molar, or between first molar and second molar of posterior teeth, were treated following the protocol. After 6 months, patients were examined using flosses. Two of the patients were unable to come back but reported that they did not experience food impaction after restoration through telephone. Except for two cases, the others were satisfied with the treatment, and no complication, such as gingivitis caused by restoration, was reported after the treatment. In one patient, food impaction recurred about 2 months after the surgery. From checkup, it we observed that the restoration was maintained. The surgery was repeated; however, food impaction recurred more than 1 month after the surgery. The food impaction occurred at the contact area between the upper first molar and the second molar (without the third molar), and the second molar was slightly loose. In another case, food impaction recurred 1 week after the surgery. From checkup, we found that the restoration was lost. The surgery was repeated and was a success for more than 6 months. However, the longevity of success of this method needs more evidence-based research.

## 4. Discussion

### 4.1. Key Ideals of the Strategy

The technique aim is to restore an intact and firm proximal contact with proper location and form relationships to prevent forceful interproximal wedging of food, which in turn protects interdental papilla, and is not to adhere the two teeth together like splint. We performed the procedure using flowable composite resin or composite resin cement with the aid of a cerclage wire under tension to rebuild the contact area.

### 4.2. Tips to Achieve a Successful Technique

#### 4.2.1. Case Selection

Loose tooth was the absolute contraindication. In this situation, a prosthodontic crown should be selected by splint. A larger mesiodistal distance of the contact area indicates a more difficult technique. In a patient with habitual food impaction at the contact area between the upper first molar and the second molar (without the third molar), the result is questionable. The result may be due to the loose maxillary bone and hence cannot endure the occlusal force leading to frequent movement of the second molar.

Because the resin adhesive system has limited adhesive strength to metal or aluminum/zirconium ceramics, this procedure were not recommended for use in the situation that food impaction involved teeth between metal prosthesis and metal prosthesis, metal prosthesis and aluminum/zirconiumprosthesis, aluminum/zirconium ceramic prosthesis and aluminum/zirconium ceramic prosthesis.

#### 4.2.2. Key Step of the Technique

For the technique to be successful, the tensile stress must be enforced through the cerclage wire to separate the contact area and squeeze the acid agent and paste of composite resin into the contact area. Another tip is to completely remove the acid-etch agent from the contact area, and adequate water rinsing is also required; otherwise, the technique will be unsuccessful. Finally, before resin setting, the tension stress must be relieved; otherwise, if resin setting was conducted under tensile stress with the cerclage wire, the tooth will enforce a mesiodistal lateral force after resin setting, resulting in trauma to the periodontal ligament and tooth migration and finally resulting in the proximal contact being unstable.

A good proximal contact is important for a well functioning dentition. Some techniques to improve the quality of a proximal contact have developed for Class II direct composite resin restoration, such as a sectional matrix system combined with a separation ring (Palodent) and a circumferential matrix system in combination with a retainer (Tofflemire): a precontoured sectional matrix system (Palodent matrix bands) combined with the separation ring, a precontoured circum-ferential matrix system (Hawe Contoured Tofflemire-Bands) without separation rings, and contact Matrix system (Danville Materials). Use of the sectional matrix system gets a tighter proximal contacts than the use of the circumferential matrix system [5, 6]. In our study, the abovementioned technique cannot be used in our clinical cases because mesiodistal distance of the contact area was very small. We used the wire that acts as a matrix and separation device at the same time. However, our aims were to squeeze the agents into the contact area and construct the form of the contact area. Thus, we emphasized that the tension stress must be relieved before resin setting. A previous study reported that the proximal contacts of a posterior composite resin restoration, which are stronger compared with those before treatment, tend to diminish after a 6-month period, whereas the proximal contacts, which are weaker compared with those before treatment, remain almost unchanged after a 6-month period [5, 6]. Thus, we reject the proposal of restoring the contact areas tighter than it used to be.

### 4.3. Advantage and Disadvantage of the Technique

The advantage of the technique is that it is very simple, atraumatic, and economic. The result is immediate, and the patient is satisfied. Even if the procedure fails, it can be carried out repeatedly without trauma to the sound tooth tissue.

Usually, adjacent teeth, such as the adjacent teeth between natural tooth and natural tooth and silica-based ceramic prosthesis and silica-based ceramic prosthesis, will become connected with each other after performing this method. After restoration, dental floss cannot be used at this situation. Of course, interdental tooth brush can be the candidate for the interdental hygiene care. However, we may modify this technique by applying a small amount of petroleum jelly on one side of a thin strip and passing it through the contact area. Therefore, one of the teeth will not be sensitive for the adhesion procedure. This allows the use of dental floss and reduces the possibility of the restoration fracture.

On the other hand, for their low viscosity, flowable composites or resin cements were selected for the restoration. Both materials have a relatively low filler content making them sensitive for wear and fracture. An adhesive connection between teeth will produce stress when teeth are loaded and lead to uncontrolled fracture. It was one of the main reasons of failure of the method and limited the longevity of success. In particular, the use of a resin cement is much concerned, as this material is not designed for restorative procedures. However, the resin cement used in our study belongs to cone building composite resin cement (see Section 2). It has relatively high strength. In the future, we will use some new flowable composites to restore the contact zone. These new flowable composites have higher filler content which makes them stronger and more wear resistant, such as Majesty Flow and Grandio Flow. Or we may modify the procedure by using the flowable composites as a surface lubricant but not curing it directly; then, a normal high strength composite resin is injected directly afterwards. We expect this method may improve the strength of the adhesive composite restoration, therefore, to improve the longevity of success.

Another issue is that the shape of the wire will leave an impression in the composite and might promote food retention. It can be improved by fine finishing and polishing the restoration.

Certainly, this is only primary results, and the evaluation period is rather short. We need a thorough study and evaluation.

## 5. Conclusion

The proposed technique is a simple, atraumatic, and economic therapeutic measure that used flowable composite resin or composite resin cement to restore vertical food impaction with the aid of cerclage wire under tension stress. As an auxiliary measure for treating vertical food impaction, the technique is useful for challenging clinical cases.
